# Editorial: Exploring the Growing Role of Cyanobacteria in Industrial Biotechnology and Sustainability

**DOI:** 10.3389/fmicb.2021.725128

**Published:** 2021-07-13

**Authors:** David J. Lea-Smith, Tina C. Summerfield, Daniel C. Ducat, Xuefeng Lu, Alistair J. McCormick, Saul Purton

**Affiliations:** ^1^School of Biological Sciences, University of East Anglia, Norwich, United Kingdom; ^2^Department of Botany, University of Otago, Dunedin, New Zealand; ^3^Michigan State University-Department of Energy (MSU-DOE) Plant Research Laboratory and Department of Biochemistry and Molecular Biology, Michigan State University, East Lansing, MI, United States; ^4^Shandong Provincial Key Laboratory of Synthetic Biology, Qingdao Institute of Bioenergy and Bioprocess Technology, Chinese Academy of Sciences, Qingdao, China; ^5^School of Biological Sciences, SynthSys and Institute of Molecular Plant Sciences, University of Edinburgh, Edinburgh, United Kingdom; ^6^Department of Structural and Molecular Biology, University College London, London, United Kingdom

**Keywords:** cyanobacteria, biotechnology, synthetic biology, protein turnover, chemical production

## Introduction

A major challenge of the 21st Century is the development of innovative, sustainable biotechnologies able to replace fossil fuel derived synthetic routes for production of bulk chemicals and high-value materials. Potentially, this challenge could be met in part through the use of cyanobacteria, the only prokaryotes capable of oxygenic photosynthesis, as microbial hosts for development of next generation industrial biotechnologies. The production of cyanobacterial biomass and synthesis of bioproducts does not require arable land, thereby avoiding competition with food production. Minimal nutrients are needed for cyanobacterial growth, and many species can be cultured in seawater, avoiding use of limited freshwater supplies. In addition, cyanobacteria can synthesize a range of high-value commercial compounds e.g., healthcare relevant pharmaceuticals, nutraceuticals, and industrial materials (Ducat et al., 2011), and their derived photo-production technology has been industrialized in several countries, including the USA, China, and Japan (Zahra et al., 2020).

However, expanding the use of cyanobacteria for production of a wider range of compounds is still restricted by multiple factors. These include the naturally slow growth rate and low biomass accumulation of most cyanobacterial species, although the recent discovery of several species, such as *Synechococcus* sp. PCC 11901 (Wlodarczyk et al., 2020), with doubling times as short as 2 h, is a promising development. The challenges of low cost culturing in outdoor photobioreactors, development of strains capable of synthesizing commercial quantities of the desired compound, and a lack of low-cost and sustainable methods for compound extraction also impede commercialization of cyanobacterial biotechnology. Strain development is hampered by genetic tools that are less developed than those available for established heterotrophic platforms such as *Escherichia coli* and *Saccharomyces cerevisiae*. Finally, our understanding of many core physiological and biochemical processes in cyanobacteria, even in the most widely studied model cyanobacterium, *Synechocystis* sp. PCC 6803 (6803), is incomplete (Mills et al., 2020).

The publications in this special issue address many of these considerations. In terms of strain selection for biotechnology applications, many factors need to be considered including growth and biomass accumulation, long term storage of strains at −80°C, growth in seawater and genetic tractability. Jeong et al. sequenced a new *Synechocystis* species, PCC 7338 (7338), which can be cultured in seawater, and compared it to multiple freshwater species, including 6803. Although the majority of genes were conserved in all the species examined, a number were unique to 7338 and likely involved in salt tolerance. Shaw et al. examined 26 photosynthetic co-cultures (consisting of a cyanobacterium and associated heterotrophic microbes) collected from a range of geographical locations and different ecosystems, focusing upon extreme environments such as hot springs and Antarctic ponds. Sequence analysis of these strains highlighted the diversity of species surviving within these extreme conditions, and provides potential opportunities for discovery of new proteins and biotechnologically-valuable compounds.

A greater understanding of cyanobacterial biology will aid commercialization of cyanobacterial biotechnology. Karlsen et al. examined why protein levels are relatively constant in 6803 subjected to artificial day-night cycles, despite the transcriptional profile of many genes altering under these conditions. Their data demonstrates that slow protein turnover and not translational regulation is the main factor in controlling protein amounts. These results have ramifications for synthetic biology and that controlling the abundance of heterologous proteins not only has to take into account expression but also degradation of the target protein. Thirumurthy et al. investigated the role of type IV pili in extracellular electron transport in 6803. This process may play a role in photoprotection by exporting excess electrons but can also be exploited for electricity production using biophotovoltaic devices, a type of microbial fuel cell (McCormick et al., 2015). Using pili-deficient mutants, their data shows that deleting these appendages has no effect on electron export, suggesting that other compounds, likely soluble electron carriers, may perform this role.

Development of new molecular tools, such as CyanoGate, a modular Golden Gate cloning kit (Vasudevan et al., 2019), aids research into understanding cyanobacterial biology and the engineering of strains for biotechnology applications. Gale et al. described the development of a CyanoGate-compatible dual reporter system to quantify and compare the efficiency of transcriptional terminators within and between species. A suite of 34 terminators were characterized and five were identified with high efficiencies in 6803, *Synechococcus elongatus* UTEX 2973 and *E. coli*. Zhang et al. utilized knock out/down and overexpression strategies to characterize twelve genes potentially involved in cell division and/or elongation in *Synechococcus elongatus* PCC 7942. Their work has advanced our understanding of these processes and identified several new targets for engineering cell morphology in cyanobacteria.

The remaining publications focus on engineering cyanobacteria for development of efficient strains more suitable for biotechnology or for higher production of a range of compounds. Wang et al. detail in their comprehensive review the recent progress in manipulating cyanobacteria for production of a range of compounds, including fatty acids, alcohols, hydrocarbons, and fatty acid esters. Song et al. analyzed the effect of minimizing photorespiratory carbon losses by expression of the formate-tetrahydrofolate ligase in 6803. Strains accumulated higher amounts of photorespiratory intermediates but had altered regulation of the carbon/nitrogen ratio. This paper highlighted both the robustness of cyanobacteria as chassis for heterologous protein expression and the value of empirical experimentation in evaluating its impact. Wang et al. engineered 6803 for production of *myo*-inositol, a compound of interest to the food and pharmaceutical industries. Introduction of the *S. cerevisiae myo*-inositol-1-phosphate synthase gene and overexpression of native genes encoding putative *myo*-inositol-1-monophosphates, together with the re-direction of carbon flux into production of the precursor compound, glucose-6-phosphate and the optimization of cultivation conditions, resulted in production of 12.72 mg L^−1^.

Overall, the work published in these studies will contribute to the development of cyanobacteria for biotechnology applications, resulting in more sustainable industries for production of a range of chemicals currently derived from agricultural or fossil fuel sources.

## Author Contributions

DL-S wrote the first draft. All authors developed the overall concept, provided input, and comments to the draft. All authors contributed to the article and approved the submitted version.

## Conflict of Interest

The authors declare that the research was conducted in the absence of any commercial or financial relationships that could be construed as a potential conflict of interest.
